# Stability and Validity of Self-Reported Depression and Anxiety in Autistic Youth

**DOI:** 10.1007/s10803-024-06456-6

**Published:** 2024-07-13

**Authors:** Soo Youn Kim, Luc Lecavalier

**Affiliations:** 1 Department of Psychology, The Ohio State University, Columbus, OH USA; 2 Nisonger Center, The Ohio State University, Columbus, OH USA

**Keywords:** Autism, Self-report, Reliability, Validity, Anxiety, Depression

## Abstract

The aim of this study was to assess test-retest reliability and diagnostic validity of self-report instruments of depression and anxiety in autistic youth. Participants were 55 autistic youth aged 8–17 years presenting with depressive or anxiety symptoms. They were interviewed with the Kiddie Schedule for Affective Disorders and Schizophrenia for School-Age Children (K-SADS-PL) and completed the Children’s Depression Inventory, Second Edition – Self Report Short (CDI 2:SR[S]) and the Revised Child Anxiety and Depression Scale (RCADS) twice, separated by a two-week interval. Test-retest reliability was measured with intraclass correlation coefficients (ICCs), and diagnostic validity was assessed using receiver operating characteristic (ROC) curves with the summary ratings on the K-SADS-PL as the criterion. The effect of participant characteristics was analyzed through a moderation analysis. Generalized anxiety (GAD) and social anxiety disorder (SOC) were the two most prevalent disorders in the sample. Test-retest reliability for most of the subscales was good (ICC = 0.74 − 0.87), with the exception of the RCADS obsessive-compulsive disorder (OCD) and GAD. The Adaptive Behavior conceptual score was a significant moderator of the reliability of the CDI 2:SR[S]. The ROC analysis suggested the RCADS SOC and the CDI 2:SR[S] to be good screening tools with inadequate specificity when appropriately sensitive cutoff scores are used. Optimal cutoff scores in this sample were lower than originally published. The findings suggest that autistic youth can provide stable reports of anxiety and depressive symptoms over time. Diagnostic validity varied according to the construct and instrument.

Autism spectrum disorder (ASD) is a neurodevelopmental disorder characterized by persistent impairment in reciprocal social communication and interaction across multiple contexts, and restricted, repetitive patterns of behavior, interests, or activities (American Psychiatric Association [APA], 2022). Studies suggest that between 55 and 94% of autistic individuals have at least one co-occurring psychiatric disorder (See Hossain et al., 2020). The prevalence of anxiety disorders in clinical and epidemiological samples reported in the scoping review by Vasa and colleagues (2020) ranged from 20 to 35% and the prevalence of depressive disorders was estimated to be between 11 and 23% (Hollocks et al., 2019; Hudson et al., 2019; Lai et al., 2019), which are both higher than the prevalence reported in the general child and adult population in the United States (Bitsko et al., 2022; Brody et al., 2018).

## Phenomenology of Internalizing Symptoms in ASD

The study of internalizing disorders in ASD has been complicated by their phenomenology. Indeed, the presentation of depression often seems to overlap with the social, cognitive, and communicative impairments that characterize ASD (Magnuson & Constantino, 2011; Pezzimenti et al., 2019; Stewart et al., 2006). For example, social withdrawal and abnormal speech patterns in ASD may be confused with fatigue or psychomotor retardation, and frequently associated symptoms such as sleep difficulties, food selectivity, and poor adaptive skills may mimic depression (Pezzimenti et al., 2019; Stewart et al., 2006). Likewise, the similarities in presentations and close associations between anxiety and core ASD symptoms are well-documented (Lecavalier et al., 2014; Wigham & McConachie, 2014). For instance, insistence on sameness, one of the core symptoms of ASD, is also commonly observed in those with anxiety and closely related to intolerance of uncertainty (Jenkinson et al., 2020). Moreover, sensory processing abnormalities have been identified to have a complex relationship with intolerance of uncertainty, anxiety, and restricted and repetitive behaviors in ASD (Hwang et al., 2020; Neil et al., 2016; South & Rodgers, 2017; Wigham et al., 2015).

In addition to the similarities and the associations between internalizing symptoms and autistic traits, accumulated evidence suggests atypical ASD-specific psychiatric symptoms that do not neatly fit in the DSM-5 framework yet elicit clinically significant level of distress (Chandrasekhar & Sikich, 2015; Kerns et al., 2014; Stewart et al., 2006). When depressed, autistic children may experience distinct symptoms such as exacerbation of self-injury and aggression, change in stereotypic behaviors or restricted interests, or even catatonia (Magnuson & Constantino, 2011; Perry et al., 2001; Stewart et al., 2006), and unique patterns of symptoms within the context of the DSM-5 such as prominent anhedonia (Kim & Lecavalier, 2021) and insomnia and restlessness (Montazeri et al., 2020). Similarly, in a series of studies, Kerns and colleagues (Kerns et al., 2014, 2017, 2021; Kerns & Kendall, 2012) reported that ASD-specific symptoms such as circumscribed worries, social fearfulness without fear of negative evaluation, distress related to change in routine, and unusual phobias are prevalent (Lau et al., 2020). Due to the complex interaction of the diagnostic overlap and the distinct presentations of internalizing disorders in the context of ASD, it is a conundrum whether the anxiety or depressive symptoms reflect a true separate co-occurring condition, an extension of varied ASD presentations, or a co-occurring condition not independent of ASD (Kerns & Kendall, 2012; Lecavalier et al., 2014; Wood & Gadow, 2010).

Due to such a complex clinical picture of depression and anxiety in ASD, relying on measures which were not developed for autistic individuals can be problematic as they may not take into consideration the unique phenomenology in ASD. In addition, previous reviews indicated that the psychometric properties of common measures are poorly understood, especially when it comes to self-reported instruments (Kim & Lecavalier, 2021, 2022; Wigham & McConachie, 2014).

## Potential Utility of Self-Report Measures

Historically, psychiatric assessments of individuals with ASD have relied heavily on informants despite the long-standing issue of informant discrepancy and the importance of multi-informant data in clinical decision making (See De Los Reyes & Epkins, 2023). The tendency to rely on informants was also an artifact of concerns regarding the accuracy of self-report measures in autistic individuals, especially in describing internal states (Kinnaird et al., 2019; Mazefsky et al., 2011). Recent studies, however, have shown that autistic individuals may be more capable of reporting their internal state than previously understood. For instance, studies examining the relationship between self-report and biological measures in autistic individuals revealed promising results (e.g., Keith et al., 2019; Rosen & Lerner, 2018; Sapey-Triomphe et al., 2019). Keith and colleagues (2019) found significant associations of self-reported but not parent-reported anxiety and auditory sensitivity with autonomic arousal at rest and autonomic reactivity during an aversive noise task. Similarly, Rosen and Lerner (2018) found that greater self-reported social anxiety was associated with an increased neural response to errors. Despite such evidence suggesting the potential utility of self-report measures in autistic youth, only a few studies have assessed important measurement properties in the context of psychiatric assessments (See Kim & Lecavalier, 2022).

## Psychometric Properties of Self-Report Measures

Kim and Lecavalier (2022) recently reviewed the literature on the psychometric properties of self-reported psychiatric tools in ASD. The vast majority of studies (28/35) focused on parent-child agreement with a pooled effect size of *r* = .42 for anxiety suggesting autistic individuals and their parents agree on anxiety symptoms just as much as neurotypical individuals and their parents do (Achenbach et al., 1987; Grills & Ollendick, 2002; Phares et al., 1989). However, far fewer studies assessed other psychometric properties such as test-retest reliability or diagnostic validity, and most of the studies that did were of low quality. There were no studies that were rated as strong that examined short-term test-retest reliability, limiting greatly our understanding of whether or not autistic individuals can provide consistent responses over short periods of time (e.g., Schiltz et al., 2017; Sharpley et al., 2019). Nine studies assessed diagnostic validity, mostly reporting sensitivity and specificity lower than indicated in the literature. Lower optimal cutoffs than originally suggested in the general child or non-ASD population were reported for self-report instruments (e.g., Carruthers et al., 2020; Mazefsky et al., 2011).

In addition to the paucity of studies establishing psychometric properties, the field has a limited understanding of the moderators of these psychometric properties of self-reported instruments. For example, although IQ is often hypothesized as a key moderator of the accuracy of instruments, there are insufficient data to reach conclusions due to frequent exclusion of individuals with intellectual or language impairment (Kim & Lecavalier, 2022). Existing studies have focused on the moderator of parent-child agreement with findings suggesting higher level of functioning associated with better informant agreement in internalizing symptoms (Burrows et al., 2018; Kaat & Lecavalier, 2015; Ooi et al., 2016; Stratis & Lecavalier, 2015).

## The Current Study

There is a need to assess the psychometric properties of self-report anxiety and depression instruments in autistic youth. Specifically, the primary aims of this study were to investigate the test-retest reliability and diagnostic validity of commonly used measures of anxiety and depression. The Children’s Depression Inventory, Second Edition – Self Report Short (CDI 2: SR[S]) and the Revised Children’s Anxiety and Depression Scale (RCADS) were selected as they are two of the most commonly used measures in research for both general child and autistic population. The Kiddie Schedule for Affective Disorders and Schizophrenia for School-Age Children (K-SADS-PL) was used to support a gold standard clinical diagnosis. Based on the literature, we hypothesized at least moderate test-retest reliability (i.e., ICC > 0.50) and lower cutoff scores than originally published for optimal sensitivity and specificity. Secondary aims were to evaluate whether age, cognitive ability, and adaptive behavior conceptual skills impact test-retest reliability, and to examine the symptom endorsement of anxiety and depression in ASD.

## Methods

### Participants

Participants were 55 children and 53 parents (children age in years range = 8–17, *M* = 13.7, *SD* = 2.7). There were two sets of sibling participants (i.e., four children with two parents). All youth had a previous diagnosis of ASD by a licensed professional, which was confirmed by the research team by reviewing the original documentation, and a score of 10 or higher on the Social Communication Questionnaire – Current version (SCQ – Current; Berument et al., 1999). All youth reportedly experienced at least a mild level of anxiety or depressive symptoms and had a standard score above 50 on the Kaufman Brief Intelligence Test – Second Edition (KBIT-2; Kaufman & Kaufman, 2004) brief IQ *or* on the adaptive behavior (AB) conceptual domain score on the Adaptive Behavior Assessment System – Third Edition (ABAS-3; Harrison & Oakland, 2015). We deemed this as the minimum level to understand simple questions/statements on the self-report questionnaires. We also selected this criterion to include youth with a wide range of levels of functioning.

### Procedure

Participants were recruited through university clinics, a tertiary diagnostic center of a children’s hospital, and online databases. Participants were offered to choose between in-person appointments at the university clinic or virtual appointments via an online teleconference platform due to the ongoing COVID-19 restrictions. Most of the data were collected virtually (*n* = 49), however six children and five parents participated in person. For virtual data collection, participants were required to have access to the internet, a quiet space, an electronic device with at least a 9.7” screen, a web camera, a microphone, and a speaker or a headphone. All measures were presented visually and were read aloud by the experimenter. Participants had two separate visits, approximately two weeks apart.

### First Assessment (Time 1)

The first visit consisted of administration of a cognitive test, completion of self-report instruments with youth, a structured interview with the child and parent, and completion of the demographics form and adaptive behavior scale by the parent. Time 1 lasted approximately 1.5–2 h. Parents could remain in the room with the child, but any assistance with the assessment protocol beyond technical help was not allowed.

### Second Assessment (Time 2)

The second appointment was held approximately 2 weeks after from the first one (*M*_*interval*_ = 15.0 days, *SD*_*interval*_ = 2.5 days, range = 11–23 days) and lasted 15–30 min for most participants. The purpose of this visit was to gather test-retest data for the self-report measures.

### Guidelines for Preventing Fraudulent Participants

To ensure integrity of virtual data collection, the following guidelines were used. First, the documentation of participant’s previous professional evaluation was thoroughly reviewed for its legitimacy (e.g., name of the agency, name and credential of the professional, tests administered, matching demographic characteristics for the participant). Second, the participants were required to keep the camera on for the entire visit. Finally, major inconsistencies on patient demographic characteristics, background information, developmental history, or reported symptoms between the provided documents for study eligibility and parent or child’s accounts of their own experiences were reviewed.

### Measures

#### Demographics Form

This form requested information about the child’s age, sex, gender, race/ethnicity, school placement, current psychiatric diagnoses, medications, and treatments along with parent’s age, sex, race/ethnicity, annual household income, and highest level of education.

#### Social Communication Questionnaire – Current (SCQ – Current)

The SCQ – Current (Berument et al., 1999) is a 40-item parent-report checklist of current symptoms of autism derived from the Autism Diagnostic Interview - Revised (ADI-R; Rutter et al., 2003). Berument et al. (1999) initially established a cutoff score of 15, however a number of studies have suggested using a lower cutoff to maximize sensitivity and specificity (e.g., Allen et al., 2007; Norris & Lecavalier, 2010; Schendel et al., 2012; Wiggins et al., 2015). This study used a cutoff score of 10, as suggested by Barnard-Brak and colleagues (2016).

#### Kaufman Brief Intelligence Test, Second Edition (KBIT-2)

The KBIT-2 (A. S. Kaufman & Kaufman, 2004) is a brief measure of verbal and nonverbal intelligence used with individuals of age 4 through 90 years. The Verbal scale measures receptive vocabulary, general knowledge, and expressive reasoning. The Nonverbal scale measures understanding of relations between concrete or abstract stimuli. The KBIT-2 yields a Verbal Standard Score, a Nonverbal Standard Score, and an IQ Composite (*M* = 100, *SD* = 15, range = 40–160). The KBIT-2 was not standardized for telepractice assessment, but it provides an online stimulus book to aid the administration process. Evidence supports score equivalence between in-person and telepractice administration modes (A. S. Kaufman & Kaufman, 2022).

#### Adaptive Behavior Assessment System, Third Edition (ABAS-3) Conceptual Domain

The ABAS-3, Parent Form, Ages 5–21 (Harrison & Oakland, 2015) measures adaptive behaviors in multiple settings for individuals of age 5 to 21 years. The instrument consists of items on a scale of 0 to 3, 0 being “Is not able to perform this behavior” and 3 being “Always or almost always performs the behavior when needed”. The ABAS-3 showed excellent internal consistency (*α* = 0.94 – 0.99) and good test-retest reliability (*r* = .80 – 0.86) in the standardization and the mixed clinical sample (Harrison & Oakland, 2015). In the current study, the three skill areas (i.e., Communication, Functional Academics, and Self-Direction) that comprise the Conceptual domain were used.

#### Children’s Depression Inventory, Second Edition – Self Report Short (CDI 2:SR[S])

The CDI 2:SR[S] is a 12-item self-report inventory assessing depressed mood in children and adolescents of age 7 through 17 years (Kovacs, 1992; Kovacs & MHS Staff, 2011). Respondents are asked to choose one description out of three that best fits how they have been feeling over the past 2 weeks (e.g., “I am sad once in a while”; “I am sad many times”; “I am sad all the time”). Responses are scored on a scale of 0 to 2, with total scores ranging between 0 and 24. The CDI 2:SR[S] showed acceptable internal consistency (*α* = 0.82), excellent test-retest reliability across a 2- to 4-week interval (obtained *r* = .77, corrected *r* = .92), and accurately classified depression compared to matched controls (Sensitivity 84%, Specificity 77%) or other clinical cases (Sensitivity 73.8%, Specificity 68.5%; Kovacs & MHS Staff, 2011). A *T*-score of 65 or higher is suggested by authors as indicative of elevated number of depressive symptoms. Both the previous and the current versions of the CDI have been used in multiple studies with autistic youth (see Kim & Lecavalier, 2021). To our knowledge, however, when it comes to the short versions, the current version has not been used in ASD (e.g., Greenaway & Howlin, 2010; Mazefsky et al., 2011).

#### Revised Children’s Anxiety and Depression Scale (RCADS)

The RCADS (Chorpita et al., 2000, 2005) is a self-report questionnaire, consisting of 47 items distributed along six subscales including separation anxiety disorder (SAD), social phobia (SOC), generalized anxiety disorder (GAD), panic disorder (PD), obsessive compulsive disorder (OCD), and major depressive disorder (MDD). It is scored on a four-point Likert scale (0 “never” – 3 “always”). Total raw scores range from 0 to 141, and *T*-scores are generated based on sex and grade level. In the initial study by Chorpita and colleagues (2000), the RCADS showed fair 1-week test-retest reliability (0.65 – 0.80), good internal consistency (*α* = 0.71 – 0.85), and good convergent and divergent validity in a non-clinical sample. Chorpita and colleagues (2005), then in a clinical sample, found optimal cutoff scores of 11 for MDD, 10 for SOC, 7 for GAD, 5 for SAD, 5 for OCD, and 12 for PD, yielding a sensitivity ranging from 0.59 to 0.78 and a specificity ranging from 0.64 to 0.92. When using the *T*-scores, the RCADS suggest a score of 65 as a borderline clinical and a score of 70 as a clinical threshold (Chorpita et al., 2005). The RCADS has been used in multiple studies with autistic youth (see Kim & Lecavalier, 2022).

#### Kiddie Schedule for Affective Disorders and Schizophrenia for School-Age Children (K-SADS-PL)

The K-SADS-PL (J. Kaufman et al., 1997, 2016) is a semi-structured diagnostic interview that is designed to be administered in a conversational style to parent and child by the same clinician. It assesses symptoms of child psychiatric disorders according to DSM-5 criteria and includes probes to guide and illustrate ways to elicit the information necessary to score each item. Final diagnoses are based on summary ratings integrating information derived from all sources of information. The initial study by J. Kaufman and colleagues (1997) found excellent interrater reliability, fair-to-good test-retest reliability, and good concurrent validity. The K-SADS-PL has been frequently used in studies with autistic children and adolescents (e.g., Caamaño et al., 2013; Mattila et al., 2012) although how the measure performs psychometrically in this population has not been assessed. In the current study, the screen interview and the diagnostic supplements that are required to diagnose Major Depression (MDD) and Anxiety Disorders (i.e., Panic Disorder (PD), Separation Anxiety (SAD), Social Anxiety (SOC), Specific Phobia (SP), Generalized Anxiety (GAD), Obsessive-Compulsive Disorder (OCD) of the most recent version, K-SADS-PL DSM-5 November 2016 (J. Kaufman et al., 2016), were administered by the lead author, a psychology doctoral student, under the supervision of the second author, a licensed psychologist.

### Statistical Analyses

All data analyses were conducted using R. First, the prevalence and the presentation of anxiety and depressive symptoms in autistic youth was examined by the number of participants receiving an above threshold rating on each item on the K-SADS-PL.

Test-retest reliability was measured through a mixed effect, absolute agreement, single measurement intraclass correlation coefficients (ICCs) between the data from two time points (McGraw & Wong, 1996). We used the guidelines suggested by Koo and Li (2016) to interpret values (i.e., 0.5 ≤ ICC < 0.75 moderate reliability, 0.75 ≤ ICC < 0.9 good reliability, and 0.9 < ICC excellent reliability). Internal consistency was examined by measuring Cronbach’s alpha.

Diagnostic validity was assessed by examining the cutoffs using the receiver operating characteristic (ROC) analysis and measuring sensitivity, specificity, positive predictive values (PPV), and negative predictive values (NPV) when compared with the criterion, the summary ratings on the K-SADS-PL. Areas under the ROC curve (AUCs) were also calculated. For the self-report measures, both raw scores and *T*-scores were used in identifying the best cutoff. The RCADS does not generate *T*-scores above 80 and therefore a score of 80 was used when the participant obtained a score of > 80. First, the Youden Index was utilized to determine the cutoff (Fluss et al., 2005; Youden, 1950), then for the scales with an acceptable AUC value (i.e., AUC = 0.70 or higher), a range of cutoff scores were analyzed to assess the sensitivity, specificity, PPV, and NPV tradeoffs with cutoff selections. The effect of demographic variables as moderators to the test-retest reliability was examined. The data from Time 2 was regressed on data from Time 1, age, and the interaction between data from Time 1 and age. Same procedure was repeated with scores on IQ and AB conceptual.

## Results

**Fig. 1:**
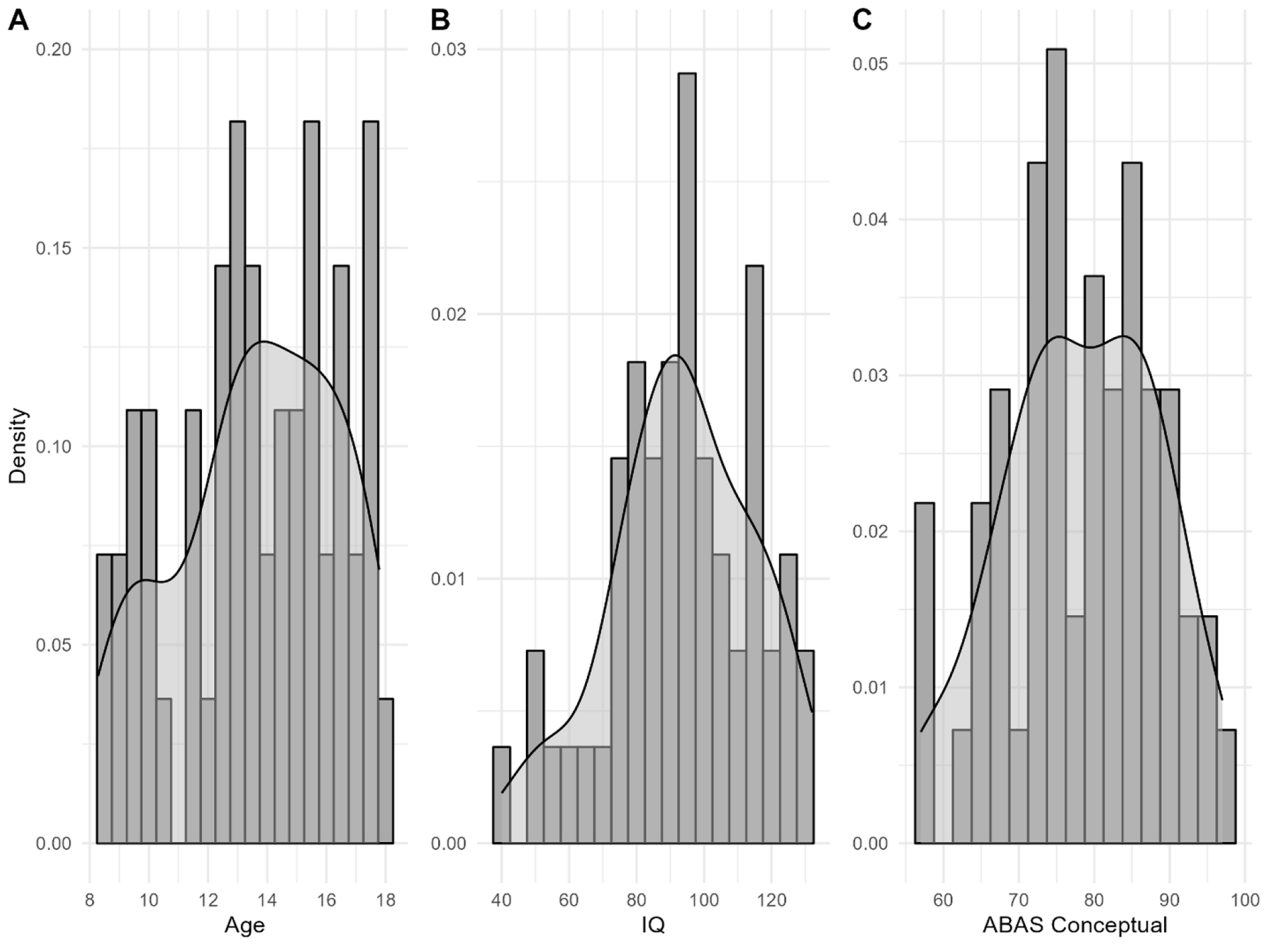
Distribution of age, IQ, and AB Conceptual scores *Note*. Each plot is a density histogram in dark grey with an overlaid kernel density curve in transparent grey. Plot A shows the distribution of age with a 0.5 year bar width. Plot B shows the distribution of IQ with a 5 standard score bar width. Plot C shows the distribution of the AB conceptual scores with a 2.5 standard score bar width

Table 1 shows demographic data for children and parents participating in the study. The final sample was characterized by a mean IQ of 93.4 (*SD* = 21.2) and a mean AB conceptual score of 78.3 (*SD* = 10.1). The majority of the sample was male (*n* = 39, 71%) and White (*n* = 43, 78%). Figure 1 depicts the distribution of participants’ age, IQ, and AB conceptual scores.

**Table 1:** Demographic characteristics of participants

	M (SD)
Age (years)	13.7 (2.7)
IQ	93.4 (21.2)
AB Conceptual Domain	78.3 (10.1)
	*n* (%)
*Child Demographics (n = 55)*	
Sex	
Female	16 (29.1)
Male	39 (70.9)
Gender	
Cisgender female	14 (25.5)
Cisgender male	38 (69.1)
Non-binary	1 (1.8)
Transgender	2 (3.6)
Race	
White	43 (78.2)
Black or African American	4 (7.3)
Asian	1 (1.8)
Multiracial	7 (12.7)
Ethnicity	
Hispanic or Latine	4 (7.3)
Non-Hispanic or Latine	51 (92.7)
Educational placement	
Regular public or private school	44 (80.0)
Regular non-inclusive classroom	9 (16.4)
Regular inclusive classroom	23 (41.8)
Regular classroom + special needs classroom	7 (12.7)
Classroom only for children with special needs	5 (9.1)
Special school for children with developmental disabilities	4 (7.3)
Home-schooled	7 (12.7)
Therapy and services	
Speech/language therapy	17 (30.9)
Occupational therapy	10 (18.2)
Physical therapy	3 (5.5)
Behavioral therapy	4 (7.3)
Counseling	27 (49.1)
Social skills group	15 (27.3)
Vocational rehabilitation	1 (1.8)
Psychotropic medication	
Yes	40 (72.7)
No	15 (27.3)
*Parent Demographics (n = 53)*	
Parent sex	
Female	50 (94.3)
Male	3 (5.7)
Annual household income	
Less than $20,000	-
$20,000 - $40,000	4 (7.5)
$40,000 - $60,000	9 (17.0)
$60,000 - $90,000	10 (18.9)
More than $90,000	30 (56.6)
Parent’s highest level of education	
Did not complete high school	1 (1.9)
High school graduate/GED	4 (7.5)
Some college/2-year degree	11 (20.8)
Bachelor’s degree	16 (30.2)
Post-grad/professional degree	21 (39.6)

### Symptom Endorsement of Anxiety Disorders and Depression

All parent-child dyads participated in the K-SADS-PL except for one youth whose parent indicated too much social anxiety. For this participant, clinical judgement was based on parent report and direct observation. Based on the K-SADS-PL summary ratings, GAD (*n* = 20, 36%) and SOC (*n* = 16, 29%) were the two most prevalent disorders, followed by MDD (*n* = 9, 16%), SP (*n* = 6, 11%), SAD (*n* = 4, 7%), OCD (*n* = 4, 7%), and PD (*n* = 1, 2%).

The item endorsements of anxiety and depression on the K-SADS-PL were examined for GAD, SOC, and MDD. Twenty-two participants completed the GAD supplement and overconcern about competence (*n* = 10), marked self-consciousness (*n* = 9) were the most endorsed types of worries, with difficulty concentrating (*n* = 19), restlessness (*n* = 17), sleep disturbance (*n* = 13), and irritability (*n* = 13) being the most frequently endorsed associated symptoms. Seventeen participants completed the SOC supplement and nearly all participants endorsed all items (i.e., social situations elicit distress, exposure almost always elicits anxiety, avoidance of social situations or endures with intense anxiety, and fears humiliation, embarrassment, or rejection). Seventeen participants completed the MDD supplement and fatigue (*n* = 8), decreased concentration (*n* = 6), and indecision (*n* = 5) were the most endorsed symptoms in addition to depressed and irritable mood.

### Reliability

Table 2 shows the test-retest and internal consistency values for the RCADS and CDI 2:SR[S]. The RCADS SAD, PD, SOC, and MDD scores and the RCADS total anxiety and internalizing scores showed good test-retest reliability with ICCs above 0.80. The RCADS OCD (ICC = 0.60), GAD (ICC = 0.65), and the CDI 2:SR[S] (ICC = 0.74) showed test-retest reliability in the moderate range. Additionally, the RCADS showed acceptable-to-good internal consistency for individual subscales (*α* = 0.74 − 0.88) whereas the CDI 2:SR[S] showed poor-to-acceptable internal consistency at Time 1 and Time 2 (*α* = 0.67 − 0.71).

**Table 2:** The RCADS and CDI 2:SR[S] T-scores, internal consistency, and the ICCs

	Time 1		Time 2		ICC [95% CI] ^a^
	T score M (SD)	Cronbach’s α	T score M (SD)	Cronbach’s α	
RCADS SAD	55.1 (12.3)	0.78	54.2 (12.8)	0.81	0.84 [0.81, 0.87]
RCADS GAD	46.1 (10.4)	0.80	42.4 (9.2)	0.78	0.65 [0.58, 0.70]
RCADS PD	52.4 (12.9)	0.87	50.0 (12.7)	0.88	0.84 [0.81, 0.86]
RCADS SOC	47.5 (10.2)	0.80	45.5 (10.3)	0.84	0.87 [0.84, 0.89]
RCADS OCD	51.9 (12.3)	0.74	49.2 (11.0)	0.74	0.60 [0.54, 0.66]
RCADS MDD	54.7 (11.5)	0.75	53.0 (11.0)	0.78	0.81 [0.77, 0.84]
RCADS Anxiety	50.7 (12.5)	0.92	47.6 (12.4)	0.94	0.86 [0.82, 0.88]
RCADS Total	51.8 (12.6)	0.93	48.8 (12.3)	0.94	0.86 [0.82, 0.88]
CDI 2:SR[S]	59.6 (11.7)	0.71	56.7 (10.2)	0.67	0.74 [0.70, 0.78]

*Note*. RCADS = Revised Child Anxiety and Depression Scale, SAD = separation anxiety disorder, GAD = generalized anxiety disorder, PD = panic disorder, SOC = social phobia, OCD = obsessive-compulsive disorder, MDD = major depressive disorder, CDI 2:SR[S] = Children’s Depression Inventory, Second Edition – Self Report Short ^a^ ICCs are calculated based on raw scores

### The Effect of Age, Cognitive Ability, and AB on Test-Retest Reliability

**Fig. 2:**
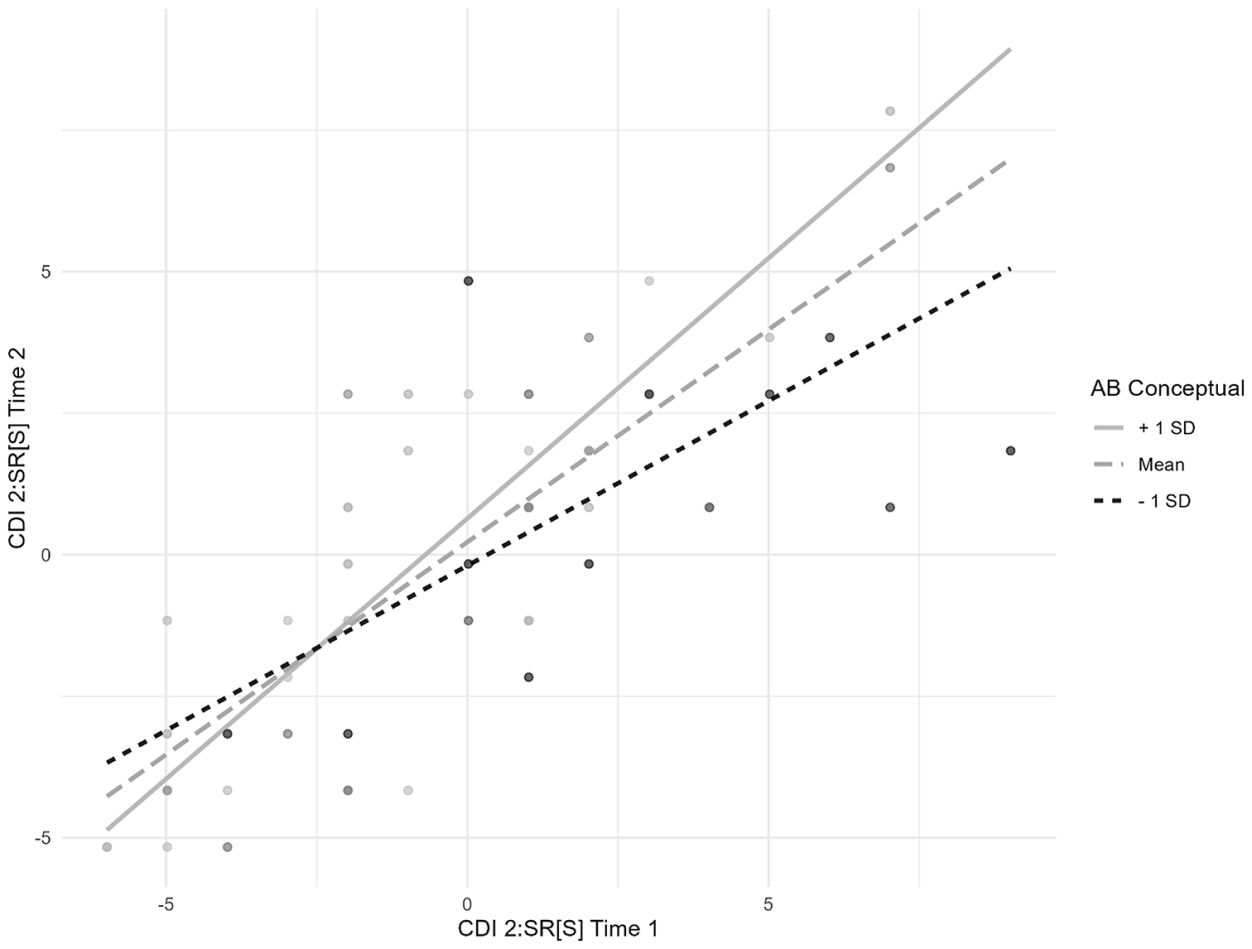
Interaction plot between the AB Conceptual and the CDI 2:SR[S] Time 1 in predicting CDI 2:SR[S] Time 2. *Note*. All variables are mean-centered

Moderation analysis identified higher AB Conceptual as a significant moderator for the CDI 2:SR[S] total scores at Time 1 predicting scores at Time 2. No other moderators were identified in predicting the association between self-reported anxiety or depressive symptoms at Time 1 and Time 2. Table 3 shows the results of the moderation analysis. Figure 2 illustrates the interaction between AB conceptual and CDI 2:SR[S] scores.

**Table 3:** The effect of demographic variables as a moderator of the test-retest reliability of the self-reported depression and anxiety symptoms

Predictors	Estimate	SE	t	*p*	95% CI
	RCADS GAD Time 2				
(Intercept)	-0.01	0.26	-0.04	0.968	-0.54–0.52
GAD Time 1	0.54	0.08	7.01	< 0.001	0.38–0.69
Age	0.18	0.10	1.86	0.068	-0.01–0.38
GAD Time 1 x Age	0.01	0.02	0.38	0.706	-0.04–0.06
*R*^*2*^ = 0.537/ *R*^*2*^ adjusted = 0.509					
	RCADS GAD Time 2				
(Intercept)	0.00	0.27	0.01	0.990	-0.54–0.54
GAD Time 1	0.55	0.08	7.21	< 0.001	0.40–0.70
IQ	0.01	0.01	0.77	0.444	-0.02–0.04
GAD Time 1 x IQ	0.00	0.00	0.43	0.671	-0.01–0.01
*R*^*2*^ = 0.508/ *R*^*2*^ adjusted = 0.479					
	RCADS GAD Time 2				
(Intercept)	0.00	0.28	0.02	0.988	-0.55–0.56
GAD Time 1	0.55	0.08	6.80	< 0.001	0.39–0.71
AB Con	-0.00	0.03	-0.16	0.874	-0.06–0.05
GAD Time 1 x AB Con	0.00	0.01	0.07	0.946	-0.02–0.02
*R*^*2*^ = 0.501/ *R*^*2*^ adjusted = 0.471					
	RCADS SOC Time 2				
(Intercept)	-0.17	0.32	-0.52	0.606	-0.82–0.48
SOC Time 1	0.80	0.06	13.26	< 0.001	0.68–0.92
Age	0.09	0.12	0.75	0.456	-0.15–0.33
SOC Time 1 x Age	0.04	0.02	1.95	0.057	-0.00–0.08
*R*^*2*^ = 0.797/ *R*^*2*^ adjusted = 0.785					
	RCADS SOC Time 2				
(Intercept)	-0.06	0.32	-0.18	0.857	-0.71–0.59
SOC Time 1	0.81	0.06	13.49	< 0.001	0.69–0.93
IQ	0.00	0.02	0.07	0.949	-0.03–0.03
SOC Time 1 x IQ	-0.00	0.00	-1.20	0.236	-0.01–0.00
*R*^*2*^ = 0.786/ *R*^*2*^ adjusted = 0.774					
	RCADS SOC Time 2				
(Intercept)	-0.00	0.33	-0.01	0.991	-0.67–0.66
SOC Time 1	0.83	0.06	13.23	< 0.001	-0.70–0.95
AB Con	0.03	0.03	1.01	0.318	-0.03–0.10
SOC Time 1 x AB Con	-0.00	0.01	-0.05	0.964	-0.01–0.01
*R*^*2*^ = 0.785/ *R*^*2*^ adjusted = 0.772					
	RCADS MDD Time 2				
(Intercept)	0.08	0.37	0.21	0.836	-0.67–0.83
MDD Time 1	0.77	0.08	9.55	< 0.001	0.61–0.93
Age	0.13	0.14	0.94	0.354	-0.15–0.41
MDD Time 1 x Age	-0.02	0.03	-0.74	0.465	-0.08–0.04
*R*^*2*^ = 0.674/ *R*^*2*^ adjusted = 0.655					
	RCADS MDD Time 2				
(Intercept)	-0.00	0.37	-0.01	0.993	-0.74–0.73
MDD Time 1	0.78	0.08	9.75	< 0.001	0.62–0.94
IQ	-0.00	0.02	-0.24	0.811	-0.04–0.03
MDD Time 1 x IQ	-0.00	0.00	-0.07	0.946	-0.01–0.01
*R*^*2*^ = 0.664/ *R*^*2*^ adjusted = 0.644					
	RCADS MDD Time 2				
(Intercept)	0.03	0.36	0.08	0.940	-0.69–0.75
MDD Time 1	0.80	0.08	10.24	< 0.001	0.64–0.96
AB Con	0.02	0.04	0.59	0.560	-0.05–0.10
MDD Time 1 x AB Con	0.01	0.01	0.87	0.388	-0.01–0.02
*R*^*2*^ = 0.673/ *R*^*2*^ adjusted = 0.653					
	CDI 2:SR[S] Time 2				
(Intercept)	-0.04	0.29	-0.15	0.883	-0.62–0.53
CDI Time 1	0.63	0.08	7.89	< 0.001	0.47–0.79
Age	0.22	0.11	2.08	0.043	0.01–0.44
CDI Time 1 x Age	0.01	0.03	0.52	0.604	-0.04–0.07
*R*^*2*^ = 0.623/ *R*^*2*^ adjusted = 0.600					
	CDI 2:SR[S] Time 2				
(Intercept)	-0.01	0.28	-0.04	0.965	-0.58–0.56
CDI Time 1	0.66	0.08	8.28	< 0.001	0.50–0.81
IQ	-0.02	0.01	-1.25	0.216	-0.04–0.01
CDI Time 1 x IQ	-0.00	0.00	-0.43	0.672	-0.01–0.01
*R*^*2*^ = 0.604/ *R*^*2*^ adjusted = 0.580					
	CDI 2:SR[S] Time 2				
(Intercept)	0.22	0.28	0.79	0.435	-0.35–0.80
CDI Time 1	0.75	0.08	9.41	< 0.001	0.59–0.91
AB Con	0.04	0.03	1.43	0.158	-0.02–0.10
CDI Time 1 x AB Con	0.02	0.01	2.24	0.030	0.00–0.03
*R*^*2*^ = 0.644/ *R*^*2*^ adjusted = 0.623					

*Note*. All entered variables are mean-centered. Estimates are unstandardized coefficients. CI = Confidence interval, GAD Time 1 = RCADS GAD raw score at Time 1, SOC Time 1 = RCADS SOC raw score at Time 1, MDD Time 1 = RCADS MDD raw score at Time 1, CDI Time 1 = CDI 2:SR[S] raw score at Time 1, AB Con = ABAS-3 conceptual domain standard score

### Diagnostic Validity

**Fig. 3:**
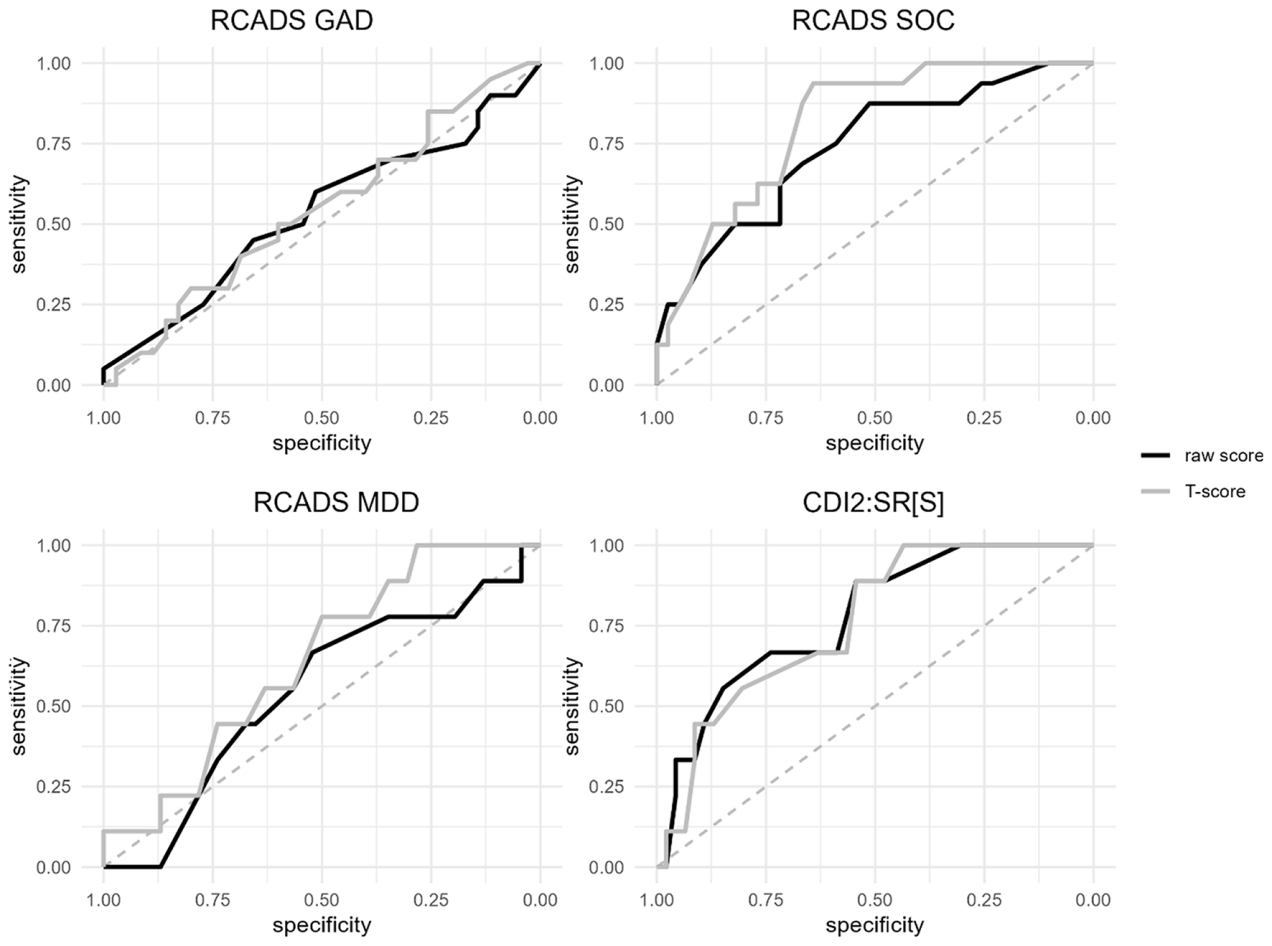
ROC curves for self-report instruments. *Note*. The top row depicts the ROC curves for the RCADS GAD on the left and the RCADS SOC on the right. The bottom row depicts the ROC curves for the RCADS MDD on the left and the CDI 2:SR[S] on the right

There were too few participants endorsing symptoms of OCD, SAD, and PD subscales of the RCADS to assess diagnostic validity (*n* = 4, 4, and 1, respectively). Table 4 shows the results based on the Youden Index for GAD, SOC, and MDD, and Fig. 3 depicts the respective ROC curves. Using clinical judgement based on the K-SADS-PL summary rating as the criterion, when raw scores were used, the Youden Index identified cutoff points of 6 for CDI 2:SR[S] (sensitivity = 0.89, specificity = 0.54), 8 for RCADS GAD (sensitivity = 0.25, specificity = 0.83), 8 for RCADS SOC (sensitivity = 0.88, specificity = 0.51), and 4 for RCADS MDD (sensitivity = 1.00, specificity = 0.13). With *T*-scores, the ROC analysis identified cutoff points of 56 for CDI 2:SR[S] (sensitivity = 1.00, specificity = 0.43), 38 for RCADS GAD (sensitivity = 0.80, specificity = 0.26), 48 for RCADS SOC (sensitivity = 0.94, specificity = 0.64), and 45 for RCADS MDD (sensitivity = 1.00, specificity = 0.28).

**Table 4:** Cutoff scores, sensitivity, specificity, PPV, NPV, and AUC values suggested by the Youden Index

	Cutoff	Sensitivity	Specificity	PPV	NPV	AUC
RCADS GAD raw score	8	0.25	0.83	0.45	0.66	0.47
RCADS GAD *T*-score	38	0.80	0.26	0.38	0.69	0.54
GAD raw score (literature)^a^	7	0.69	0.72	--	--	--
RCADS SOC raw score	8	0.88	0.51	0.42	0.91	0.74
RCADS SOC *T*-score	48	0.94	0.64	0.52	0.96	0.81
SOC raw score (literature)^a^	10	0.59	0.64	--	--	--
RCADS MDD raw score	4	1.00	0.13	0.18	1.00	0.45
RCADS MDD *T*-score	45	1.00	0.28	0.21	1.00	0.64
MDD raw score (literature)^a^	11	0.74	0.77	--	--	--
CDI 2:SR[S] raw score	6	0.89	0.54	0.28	0.96	0.78
CDI 2:SR[S] *T*-score	56	1.00	0.43	0.26	1.00	0.76
*T*-score (literature) ^b^	65	0.74	0.69	0.55	0.83	--

*Note*. RCADS = Revised Child Anxiety and Depression Scale, GAD = generalized anxiety disorder, SOC = social phobia, MDD = major depressive disorder, CDI 2:SR[S] = Children’s Depression Inventory, Second Edition – Self Report Short ^a^ Based on Chorpita et al. (2005) ^b^ Based on the clinical sample data from Kovacs & MHS Staff (2011)

The RCADS SOC and the CDI 2:SR[S] yielded an AUC value above 0.70. Table 5 presents a range of cutoff scores for these two scales. For the RCADS SOC, *T*-scores performed better than raw scores with a higher AUC value. For the CDI 2:SR[S], both raw scores and *T*-scores performed similarly. The raw score cutoff indicated by the Youden Index (raw score = 6) showed higher sensitivity and NPV and a lower specificity and PPV. A cutoff of 8, which has a lower Youden Index, was associated with more balanced metrics and higher specificity (sensitivity = 0.67, specificity = 0.74, PPV = 0.33, NPV = 0.92). The *T*-score indicated by the Youden Index (*T*-score = 56) yielded a perfect sensitivity and NPV, yet much lower specificity and PPV. A slightly higher *T*-score of 60 (sensitivity = 0.89, specificity = 0.54, PPV = 0.28, NPV = 0.96) or 64 (sensitivity = 0.67, specificity = 0.63, PPV = 0.26, NPV = 0.91) was associated with higher specificity.

**Table 5:** Sensitivity, specificity, PPV, and NPV based on different cutoff scores for the RCADS SOC and CDI 2:SR[S]

Cutoffs	Sensitivity	Specificity	PPV	NPV	AUC
RCADS SOC					
Raw score					
16	0.25	0.95	0.67	0.76	0.74
14	0.50	0.82	0.53	0.80	
10	0.69	0.67	0.46	0.84	
**8**	**0.88**	**0.51**	**0.42**	**0.91**	
4	0.94	0.26	0.34	0.91	
*T*-score					
61	0.25	0.95	0.67	0.76	0.81
58	0.50	0.87	0.62	0.81	
55	0.56	0.82	0.56	0.82	
52	0.63	0.77	0.53	0.83	
**48**	**0.94**	**0.64**	**0.52**	**0.96**	
41	1.00	0.38	0.40	1.00	
CDI 2:SR[S]					
Raw score					
12	0.33	0.97	0.60	0.88	0.78
10	0.44	0.89	0.44	0.89	
8	0.67	0.74	0.33	0.92	
**6**	**0.89**	**0.54**	**0.28**	**0.96**	
4	1.00	0.30	0.22	1.00	
*T*-score					
72	0.44	0.91	0.50	0.89	0.76
67	0.56	0.80	0.36	0.90	
64	0.67	0.63	0.26	0.91	
60	0.89	0.54	0.28	0.96	
**56**	**1.00**	**0.43**	**0.26**	**1.00**	

*Note*. The cutoff scores were selected from the points on the ROC curve which made changes to all metrics compared to the previous point. The cutoff points identified through the Youden Index are bolded

## Discussion

This is the first study to examine both the test-retest reliability and diagnostic validity of self-report instruments of anxiety and depressive symptoms in autistic youth. The findings provide insight into the nature of how autistic youth report their own internalizing symptoms and into the strengths and challenges of using self-reported instruments to measure different anxiety and depressive symptoms.

### Test-Retest Reliability

Data indicated moderate-to-good level of test-retest reliability in autistic youth when reporting anxiety and depressive symptoms. Considering previous findings that autistic children’s ratings concur with their parents just as much as neurotypical children (Kim & Lecavalier, 2022), it appears as though autistic youth are, in general, reliable reporters of their inner state. Specifically, the RCADS overall showed good test-retest reliability (ICC = 0.81 – 0.87), and the CDI 2:SR[S] showed comparable test-retest reliability (ICC = 0.74) to what has been reported in the scale development study (Kovacs & MHS Staff, 2011; obtained *r* = .77). It is important to take into consideration the time-dependent nature of depression in interpreting these results. MDD, by definition, consists of a Major Depressive Episode which lasts for two or more weeks which means depending on the duration or severity of the episode, the 2-week interval implemented in this study may have been enough to see meaningful changes in the symptoms. Disentangling the temporal stability of the measurement from the stability of the construct is a complex task which future studies must contend with. Temporal stability, that is, showing that an instrument is not vulnerable to random fluctuations, is a prerequisite to measuring change.

There were exceptions to the good test-retest reliability with a few scales. The RCADS OCD scale showed the poorest reliability with an ICC of 0.60. This is in line with previous findings from the initial study by Chorpita and colleagues (2000) and studies in ASD (Kaat & Lecavalier, 2015). More research is needed on the overlapping phenomenology and the adaptation of instruments to differentiate OCD from ASD symptoms. The RCADS GAD scale also showed a relatively poorer ICC of 0.65, lower than the 0.79 originally reported by Chorpita and colleagues (2000) for a one-week interval. This inconsistency may be due to the differences in the time interval or potentially related to the poor validity observed in this study.

### Diagnostic Validity

#### Diagnostic Validity of Self-Reported Social Anxiety

The RCADS SOC showed the best performance in predicting diagnoses, in fact, better than originally reported by Chorpita and colleagues (2005). However, it is noteworthy that the identified cutoff was a *T*-score of 48. As expected, with a cutoff this low, sensitivity is much higher than specificity (0.94, 0.64, respectively), suggesting utility as a screening tool, rather than a diagnostic one. The low cutoff score suggests that contrary to parent report which often leads to an over-reporting of social anxiety due to the overlapping nature of constructs (Kaat & Lecavalier, 2015; Renno & Wood, 2013), the opposite may be true with self-report (Carruthers et al., 2020). Contrary to previous reports on atypical presentation of social anxiety (Kerns et al., 2014; Kerns & Kendall, 2012; Lau et al., 2020), experiencing fear of negative evaluation was in fact frequently endorsed by participants in this study, and therefore the absence of this fear did not appear to be the cause of the observed low cutoff score.

#### Diagnostic Validity of Self-Reported Depression

The CDI 2:SR[S] performed significantly better in predicting diagnoses than the RCADS MDD in autistic youth. A raw score cutoff of 6 and a *T*-score cutoff of 56 were identified by the Youden Index, although a slightly higher cutoff (*T*-score 60 or 64) may be more desirable in situations where a more balanced sensitivity and specificity are needed. The sensitivity and NPV of CDI 2:SR[S] were comparable but the specificity and PPV were considerably lower than previous reports (Cho et al., 2022; Kovacs & MHS Staff, 2011). On the other hand, the RCADS MDD showed poor accuracy in predicting diagnoses. This is inconsistent with previous findings from a non-ASD clinical sample as Chorpita and colleagues (2005) found the MDD scale to show the most favorable prediction above chance. The CDI 2:SR[S] differs from the RCADS in two important ways: having a time frame of two weeks instead of one and having individuals pick one sentence in a group of three instead of a Likert scale, which is known to be more challenging for children (Mellor & Moore, 2014). The discrepant results between these two measures may therefore in part be due to the structure of the measures.

#### Diagnostic Validity of Self-Reported Generalized Anxiety

The RCADS GAD showed the worst diagnostic accuracy with an AUC value close to 0.50, suggesting no discrimination power. Few participants obtained above threshold ratings on the specific K-SADS-PL items (e.g., preoccupation with past behavior, worries about future), despite GAD being the most prevalent diagnosis in our sample. This may be explained by circumscribed worries, or atypical worries related to individuals’ circumscribed interests (Kerns et al., 2014, 2021; Kerns & Kendall, 2012). In other words, the poor diagnostic validity could be related to the RCADS GAD items not encompassing enough variability in the content of the assessed worries. Future research using different instruments is needed to further determine whether poor validity with generalized anxiety exists beyond the RCADS.

### The Effect of Age, Cognitive Ability, and AB on Test-Retest Reliability

Overall, data suggested a potential relationship with AB Conceptual and the psychometric properties of depressive symptoms. Higher AB conceptual skills led to higher test-retest reliability of depressive symptoms, similar to the previously reported relationship between IQ and inter-rater reliability (Kaat & Lecavalier, 2015; Stratis & Lecavalier, 2015). We interpret this association with caution as the effect was marginal and the results should not be generalized outside of the score range of the current sample (i.e., 57 to 97). Further caution is warranted as this relationship was not observed across different measures (i.e., CDI 2:SR[S] and RCADS MDD). Further investigation is required to examine the role of age, practical and social adaptive skills, IQ, and other potential predictors of reliability and validity in self-reported internalizing symptoms using a larger sample.

### Implications

This study has several implications for clinicians and researchers. There have been few studies investigating the psychometric properties of instruments for internalizing symptoms in autistic children. To our knowledge, this was the first study to examine the test-retest reliability and the diagnostic validity of the RCADS and the CDI 2:SR[S], two widely used instruments. This is also the first study to use the K-SADS-PL as a reference standard for exploring diagnostic validity in autistic youth in the US. This study included children with a wide range of functioning which allows to examine the effect of participant characteristics on psychometric properties. The inclusion of individuals with a wide range of cognitive ability may help to decrease the selection bias inherent in limiting studies to those individuals with average to above average cognitive functioning.

### Limitations

Results of the current study should be interpreted in light of several considerations. This study was conducted with a relatively small sample of convenience. Nevertheless, it was well-powered to assess temporal stability and compared favorably to several other diagnostic validity studies. The use of the K-SADS-PL in establishing clinical diagnoses may have precluded the investigation of atypical presentations given that it is based on the DSM-5. Along these lines, only 11% of the current sample presented with a specific phobia which is inconsistent with the literature (See van Steensel & Heeman, 2017; Vasa et al., 2020; White et al., 2009). Additionally, this study did not include prior psychiatric diagnoses as moderators of psychometric properties. Prior diagnoses may affect the level of insight into participants’ current symptoms and future studies are encouraged to take this into consideration.

## Conclusions

In conclusion, findings of this study suggest autistic youth to be reliable reporters of internalizing symptoms over time. The data also question the validity of existing cutoffs. The RCADS is not recommended to be used in screening for or measuring symptoms of generalized anxiety or major depression, although it shows promise as a screening tool for social anxiety when using lower cutoffs than established for the general population. With respect to depression, the CDI 2:SR[S] may be used as a screener, but also with a lower cutoff than previously recommended. Given the variability in findings, future research is warranted to better understand the validity of autistic youth’s self-report of internalizing symptoms. Future directions in studying diagnostic validity include replicating these results with different measures, including parent-report rating scales for richer interpretation, and using both traditional and autism-specific instruments to assign diagnoses.
